# Ultrasound-based radiomics technology in fetal lung texture analysis prediction of neonatal respiratory morbidity

**DOI:** 10.1038/s41598-022-17129-8

**Published:** 2022-07-26

**Authors:** Yanran Du, Jing Jiao, Chao Ji, Man Li, Yi Guo, Yuanyuan Wang, Jianqiao Zhou, Yunyun Ren

**Affiliations:** 1 Department of Ultrasound, Ruijin Hospital, Shanghai Jiaotong University School of Medicine, No. 197, Rui Jin 2nd Road, Shanghai, 200025 China; 2 Obstetrics and Gynecology Hospital of Fudan University, No.128, Shenyang Road, Shanghai, 200090 China; 3 Department of Electronic Engineering, Fudan University, No. 220, Handan Road, Yangpu District, Shanghai, 200433 China; 4 Putuo Hospital Affiliated to Shanghai University of Traditional Chinese Medicine, No.164, Lanxi Road, Shanghai, 200062 China

**Keywords:** Randomized controlled trials, Ultrasonography

## Abstract

To develop a novel method for predicting neonatal respiratory morbidity (NRM) by ultrasound-based radiomics technology. In this retrospective study, 430 high-throughput features per fetal-lung image were extracted from 295 fetal lung ultrasound images (four-chamber view) in 295 single pregnancies. Images had been obtained between 28^+3^ and 37^+6^ weeks of gestation within 72 h before delivery. A machine-learning model built by RUSBoost (Random under-sampling with AdaBoost) architecture was created using 20 radiomics features extracted from the images and 2 clinical features (gestational age and pregnancy complications) to predict the possibility of NRM. Of the 295 standard fetal lung ultrasound images included, 210 in the training set and 85 in the testing set. The overall performance of the neonatal respiratory morbidity prediction model achieved AUC of 0.88 (95% CI 0.83–0.92) in the training set and 0.83 (95% CI 0.79–0.97) in the testing set, sensitivity of 84.31% (95% CI 79.06–89.44%) in the training set and 77.78% (95% CI 68.30–87.43%) in the testing set, specificity of 81.13% (95% CI 78.16–84.07%) in the training set and 82.09% (95% CI 77.65–86.62%) in the testing set, and accuracy of 81.90% (95% CI 79.34–84.41%) in the training set and 81.18% (95% CI 77.33–85.12%) in the testing set. Ultrasound-based radiomics technology can be used to predict NRM. The results of this study may provide a novel method for non-invasive approaches for the prenatal prediction of NRM.

## Introduction

Neonatal respiratory morbidity (NRM), associated with prematurity, is the leading cause of mortality and morbidity^1^. Fetal lung maturity (FLM) was influenced by many factors, including gestational diabetes mellitus (GDM) and pre-eclampsia (PE), the two most common complications of pregnancy^2,3^. With the increasing use of assisted reproductive technology (ART), the incidence of gestational hypertension and GDM in these women is 11.0% and 15.1% respectively^4^. Accurate estimates of fetal lung development in pregnancies during complications will help obstetricians make clinical decisions that can avoid unnecessary premature birth and ensure optimal maternal and fetal outcomes. Although the methods and techniques have been improved since the L/S ratio was applied 25 years ago, FLM detection still cannot predict whether the fetal lung is mature or not^5^.

In recent years, the combination of ultrasound images with artificial intelligence technology has provided new ideas for the detection of FLM^6,7^. Radiomics is a technology that combines big data and medical imaging-assisted diagnosis. By extracting and mining high-throughput features from multi-modality images, it can quantitatively analyze the human molecular and genetic changes hidden behind medical images. This technology has been widely used in the analysis of ultrasound images^8–10^. But to the best of our knowledge, there is no published research on ultrasound-based radiomics technology being employed to study the development of fetal lungs during pregnancy complications.

In the present study, by collecting fetal lung ultrasound standard images, the fetal lung texture characteristics were analyzed and compared using ultrasound-based radiomics technology. A neonatal respiratory morbidity prediction model was established by using the ultrasound image features of fetal lungs combined with clinical characteristics (gestational age and pregnancy complications), which may provide a new method for non-invasive prediction of NRM.

## Results

### Populations

The characteristics of the study cohort are summarized in Table 1. Included in the study were 295 standard fetal lung ultrasound images obtained within 72 h before delivery, including 210 in the training set and 85 in the testing set. In the end, there were 69 (69/295, 23.4%) newborns with neonatal respiratory morbidity, among which 49 (49/69, 71.0%) newborns with transient tachypnea of the newborn and 20 (20/69, 29.0%) with respiratory distress syndrome.

**Table 1:** Characteristics of study cohort.

Characteristic	Training set (n = 210)	Testing set (*n* = 85)
Maternal age (years)	31 ± 3.88	31 ± 4.23
GA at ultrasound (weeks)	28–37 (35 ± 2.42)	29–37 (35 ± 2.11)
**Pregnancy complications (GDM or PE)**		
With	73 (34.76%)	25 (29.41%)
Without	137 (65.24%)	60 (70.59%)
**Mode of delivery**		
Spontaneous vaginal	75 (35.71%)	52 (61.18%)
Cesarean	135 (64.29%)	33 (38.82%)
Birth weight (g)	3006 ± 562	3212 ± 616
**Sex of newborn**		
Female	116 (55.24%)	51 (60.00%)
Male	94 (44.76%)	34 (40.00%)
**5-min Apgar score**		
≤ 7	4 (1.90%)	3 (3.53%)
> 7	206 (98.09%)	82 (96.47%)
**Neonatal prognosis**		
No neonatal respiratory morbidity	159 (75.71%)	67 (78.82%)
Neonatal respiratory morbidity	51 (24.29%)	18 (21.18%)
TTN	35 (16.67%)	14 (16.47%)
RDS	16 (7.62%)	4 (4.71%)
NICU admission	51 (24.29%)	18 (21.18%)

Data are presented as mean ± SD or *n* (%).

*GA* gestational age, *GDM* gestational diabetes mellitus, *PE* pre-eclampsia, *TTN* transient tachypnea of the newborn, *RDS* respiratory distress syndrome, *NICU* neonatal intensive care unit.

### Neonatal respiratory morbidity prediction model

**Figure 1:**
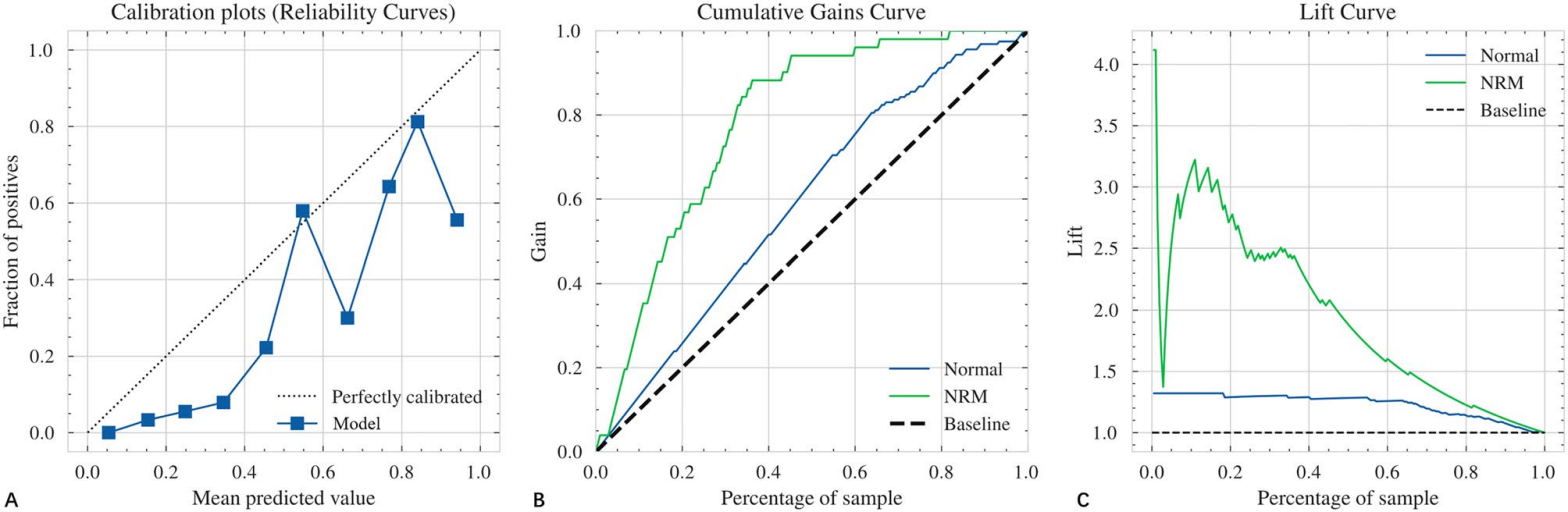
The calibration plots, gain curve and lift curve of machine-learning model built by RUSBoost architecture for predicting the possibility of NRM. (**A**) Calibration plots; (**B**) gain curve; (**C**) lift curve. *RUSBoost* random under-sampling with AdaBoost, *NRM* neonatal respiratory morbidity.

**Figure 2:**
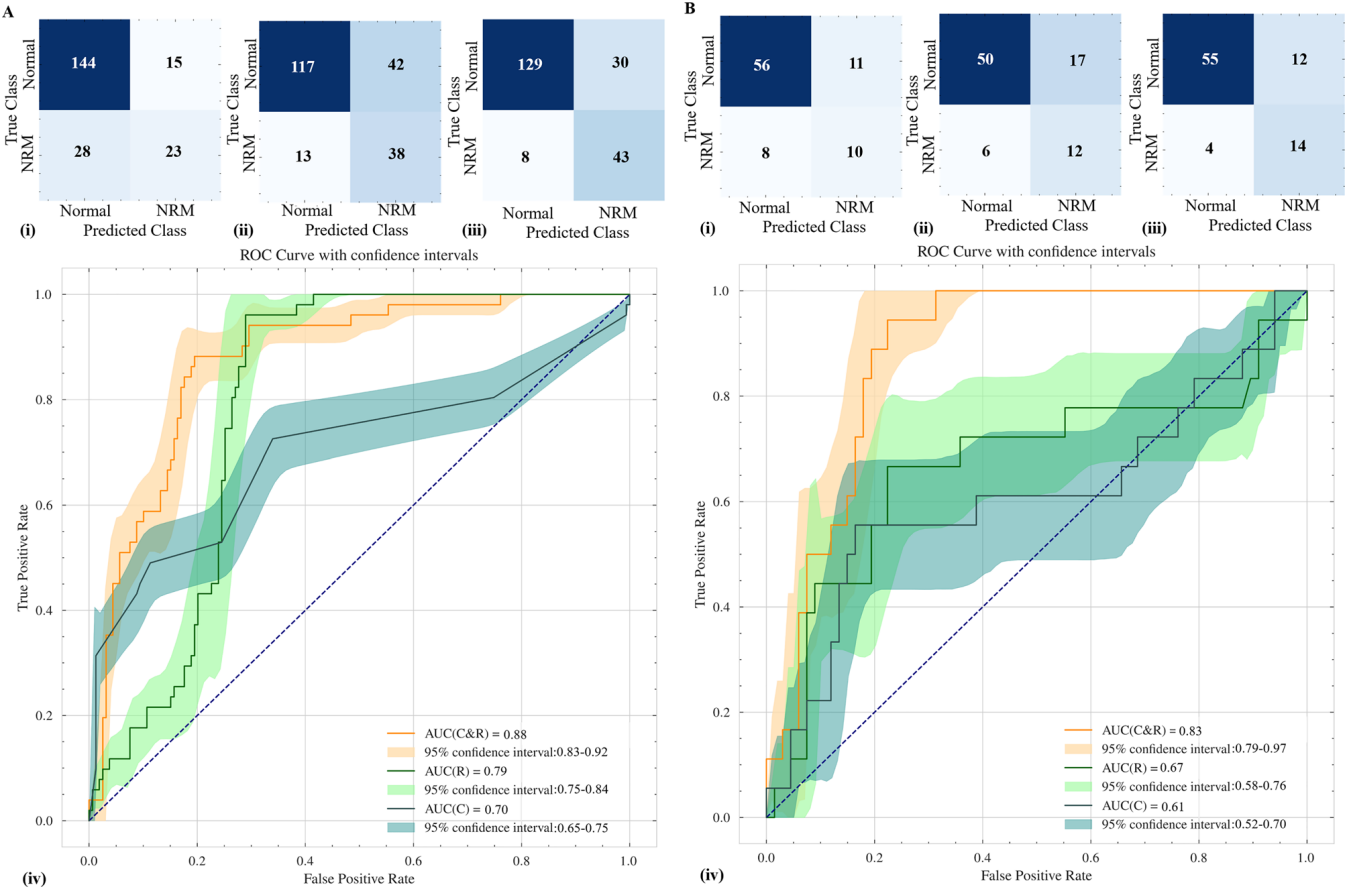
The confusion matrix and ROC curve for predicting neonatal respiratory morbidity depending on different features. (**A**) Results of the training set; (**B**) results of the testing set; (i), (ii) and (iii): confusion matrix; (iv): ROC curves. (i): Results of the model based on clinical features. (ii) Results of the model based on radiomics features. (iii) Results of the model based on the combination of clinical and radiomics features. *NRM* neonatal respiratory morbidity, *ROC* receiver operating characteristic curve, *AUC* area under the receiver-operating-characteristics curve, *C* clinical features, *R* radiomics features; *C&R* the combination of clinical and radiomics features.

By permuting out-of-bag data feature of random regression forest, 20 radiomics features and 2 clinical features (GA and Pregnancy complications) were selected and input into RUSBoost classifier to predict the possibility of NRM. Calibration, gain and lift curves created with the cross-validation results to see how much the predictive model would have helped to predict possibility of NRM are shown in Fig. 1. The confusion matrix and model performance for predicting neonatal respiratory morbidity depending on different features (clinical features, radiomics features and the combination of clinical and radiomics features) are shown in Table 2 and Fig. 2. In the testing set, the area under the receiver operating characteristic curves (AUCs) of three models were 0.61 (95% CI 0.52–0.70) in clinical model, 0.67 (95% CI 0.58–0.76) in radiomics model and 0.83 (95% CI 0.79–0.97) in clinical & radiomics model respectively. For the combination of clinical and radiomics features, the diagnostic efficacy of the neonatal respiratory morbidity prediction model achieved sensitivity of 77.78% (95% CI 68.30–87.43%), specificity of 82.09% (95% CI 77.65–86.62%), accuracy of 81.18% (95% CI 77.33–85.12%), positive predictive value (PPV) of 53.85% (95% CI 44.08–63.93%), negative predictive value (NPV) of 93.22% (95% CI 90.16–96.31%) and AUC of 0.83 (95% CI 0.79–0.97). The risk probability of NRM predicted by the clinical & radiomics model was 0.008–0.999 (0.796 ± 0.334) in NRM cases, while it was 0.001–0.999 (0.285 ± 0.268) in normal cases.

**Table 2:** Model performance for predicting neonatal respiratory morbidity depending on different features.

	Clinical features		Radiomics features		Clinical and radiomics features	
	Training set (95% CI)	Testing set (95% CI)	Training set (95% CI)	Testing set (95% CI)	Training set (95% CI)	Testing set (95% CI)
Sensitivity	45.10% (38.27–51.99%)	55.56% (43.35–67.96%)	74.51% (68.58–80.27%)	66.67% (54.91–78.86%)	84.31% (79.06–89.44%)	77.78% (68.30–87.43%)
Specificity	90.57% (88.23–92.89%)	83.58% (79.10–88.00%)	73.58% (70.11–77.02%)	74.63% (69.30–80.08%)	81.13% (78.16–84.07%)	82.09% (77.65–86.62%)
Accuracy	79.52% (76.60–82.43%)	77.65% (73.20–82.10%)	73.81% (70.80–76.74%)	72.94% (68.06–77.98%)	81.90% (79.34–84.41%)	81.18% (77.33–85.12%)
PPV	60.53% (52.29–68.82%)	47.62% (36.07–59.01%)	47.50% (42.00–52.93%)	41.38% (32.04–50.99%)	58.90% (53.25–64.51%)	53.85% (44.08–63.93%)
NPV	83.72% (80.81–86.61%)	87.50% (83.43–91.67%)	90.00% (87.46–92.47%)	89.29% (85.03–93.66%)	94.16% (92.08–96.17%)	93.22% (90.16–96.31%)
AUC	0.70 (0.65–0.75)	0.61 (0.52–0.70)	0.79 (0.75–0.84)	0.67 (0.58–0.76)	0.88 (0.83–0.92)	0.83 (0.79–0.97)

Clinical features include pregnancy complications and gestational age. Radiomics features were based on free-hand delineation of region of interests by Radiologist A.

*CI* confidence interval, *PPV* positive predictive value, *NPV* negative predictive value, *AUC* area under the receiver-operating-characteristics curve.

The intraclass correlation coefficient (ICC) values of each selected radiomics features depending on different delineations (manual delineation by radiologists A and B and square delineation) are shown in Table 4, which were 0.705 (95% CI 0.599–0.790) to 0.961 (95% CI 0.944–0.973). The diagnostic performance of the fetal-lung-texture analysis based on the ROIs delineated by radiologist B (free-hand) and on the square delineation (40 × 40 pixels) are shown in Table 3. The diagnostic performance of testing set was very similar to that of radiologist A, with AUCs of 0.87 (95% CI 0.78–0.96) (Radiologist B), 0.88 (95% CI 0.79–0.96) (square delineation) and 0.83 (95% CI 0.79–0.97) (Radiologist A), sensitivities of 74.67% (95% CI 55.41–77.93%) (Radiologist B), 76.12% (95% CI 71.07–81.05%) (square delineation) and 77.78% (95% CI 68.30–87.43%) (Radiologist A), specificities of 80.60% (95% CI 75.84–85.25%) (Radiologist B), 88.89% (95% CI 81.41–95.93%) (square delineation) and 82.09% (95% CI 77.65–86.62%) (Radiologist A) and accuracies of 77.65% (95% CI 73.23–82.00%) (Radiologist B), 78.82% (95% CI 74.48–82.98%) (square delineation) and 81.18% (95% CI 77.33–85.12%) (Radiologist A).

**Table 3:** Diagnostic performance of fetal-lung-texture analysis, based on free-hand delineation of region of interest by radiologist B and square delineation by radiologist B.

	Radiologist B free-hand delineation		Square delineation	
	Training set (95%CI)	Testing set (95%CI)	Training set (95%CI)	Testing set (95%CI)
Sensitivity	84.31% (79.33–89.19%)	74.67% (55.41–77.93%)	72.55% (66.18–78.46%)	76.12% (71.07–81.05%)
Specificity	90.57% (88.24–92.96%)	80.60% (75.84–85.25%)	94.34% (92.51–96.12%)	88.89% (81.41–95.93%)
Accuracy	89.05% (86.89–91.23%)	77.65% (73.23–82.00%)	89.05% (86.90–91.05%)	78.82% (74.48–82.98%)
PPV	74.14% (68.30–80.05%)	48.00% (37.88–57.80%)	80.43% (74.53–86.13%)	50.00% (40.97–58.66%)
NPV	94.74% (2.98–96.47%)	90.00% (86.22–93.84%)	91.46% (89.30–93.49%)	96.23% (93.67–98.64%)
AUC	0.94 (0.88–0.99)	0.87 (0.78–0.96)	0.86 (0.82–0.89)	0.88 (0.79–0.96)

*CI* confidence interval, *PPV* positive predictive value, *NPV* negative predictive value, *AUC* Area under the receiver-operating-characteristics curve.

## Discussion

The results of the present study revealed that fetal lung texture analysis by ultrasound-based radiomics technology can be used to predict the probability of neonatal respiratory morbidity by analyzing fetal lung ultrasound images and in combination with clinical characteristics (GA and pregnancy complications). It may provide a new method for noninvasive prediction of NRM.

The clinical utility of FLM assays has been largely debated^11^. At present, instead of studying several components of the amniotic fluid through amniocentesis, the application of prenatal corticoids and postnatal surfactant has become the main clinical measure to reduce neonatal respiratory diseases^12^. However, the recommended type of corticosteroid and the gestational window of treatment administration have not been clearly defined^13^. Studies have shown that there are potentially important risks of corticosteroids in neurodevelopment and fetal metabolic planning^14–16^. In a study of 278,508 live-born singletons of 24 weeks gestation or above in Finland, antenatal steroid was shown to be associated with the delivery of small fetus at birth^17^. The results of this study may provide a new method for non-invasive approaches for the prenatal assessment of FLM, which can not only avoid the fear and discomfort of amniocentesis, help to decide whether to use prenatal corticosteroids, but also refine the timing of delivery in high-risk pregnancies.

With the widespread use of ultrasound in obstetrics, several attempts have been made to evaluate fetal lung maturity noninvasively. Sm et al.^18^ showed that a measured elevated acceleration-to-ejection time ratio of the fetal pulmonary artery doppler was independently associated with the development of RDS in preterm infants and thus a possible marker of lung maturity. Attempts to quantify fetal lung volume in normal pregnancies by using 3-dimensional ultrasonography though useful in cases like diaphragmatic hernia have not been shown to objectively evaluate FLM^19,20^. In addition, gray scale measurement^21^, fetal lung tissue movement assessment^22^, and evaluation of fetal lung images relative to fetal liver and fetal placenta images^23^ have been tried to proposed as a possible tool for the assessment of fetal lung maturity. Unfortunately, the accuracy of this diagnosis is very poor, so no clinical significance is found. Recently, Palacio et al.^24^ reported that the quantitative ultrasound lung texture analysis could be used to evaluate fetal lung maturity and showed an accuracy similar to that of biochemical tests in amniotic fluid previously reported. In this study, the overall performance of neonatal respiratory morbidity prediction model based on fetal lung texture analysis by ultrasound-based radiomics technology achieved AUC of 0.83–0.88, sensitivity of 77.78–84.31%, specificity of 81.31–82.09% and accuracy of 81.18–81.90%. These ultrasound images, which appear indistinguishable to the naked eye, could quickly and accurately predict the risk of NRM in the fetus. And the images collected by different trained doctors using different machines do not affect the estimation results of the model.

Our previous research^25^ reported that there were great differences in fetal lung texture between pregnancies with GDM, PE and normal pregnancy and between different gestational ages. In our study population, there were 33.2% (98/295) of pregnant women with GDM and PE. Among these, the proportion of newborns with NRM was nearly twice that in the normal pregnancy group (6.1% vs 3.2%). Therefore, in this study, the model was established by high-throughput radiomics features and two clinical features (pregnancy complications and gestational age). Studies^26^ have shown that the accuracy and PPV of tests on amniotic fluid in predicting NRM was 73.3% (57.5–81.6%) and 27.1% (18.0–34.1%) respectively. In this study, results showed improvements by about 8.2 pp in accuracy (81.5%) and 29.3 pp in PPV (56.4%).

Our study had several limitations: First, large amounts of data are necessary in radiomics for mining concealed prognostic information and to avoid overfitting. Expanding the sample size, especially the positive sample size, would improve the stability and accuracy of the model. Second, in this study, the ROIs of fetal lungs were performed manually. A computer system will be used to identify fetal lung tissue automatically, so that the model could be used more conveniently. Third, it is a single-center study, and image acquisition and delineation were performed by highly-trained personnel. But as the number of operators and settings increases, there will be many unqualified images. Multi-center research will be carried out in the future.

In conclusion, ultrasound-based radiomics technology can be used to predict neonatal respiratory morbidity. The results of this study may provide a new method for non-invasive approaches for the prenatal prediction of NRM.

## Methods

### Patients

**Figure 3:**
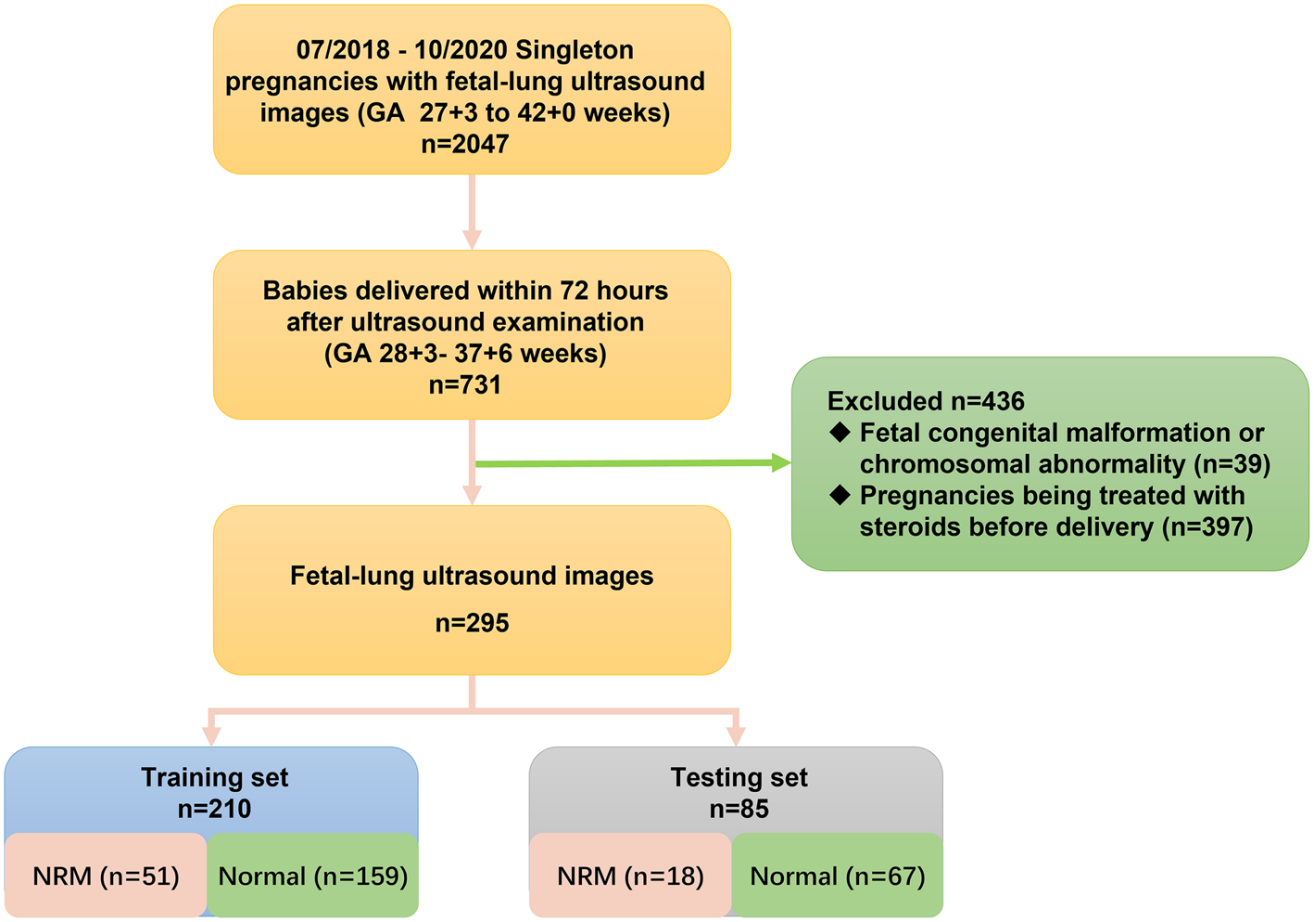
Flowchart of the selection of the study population. *NRM* neonatal respiratory morbidity.

Between July 2018 and October 2020, 2047 routine fetal-lung ultrasound images (either right or left lung) from 2047 women with singleton pregnancy were obtained, at gestational ages (GA) ranging from 27^+3^ to 42^+0^ weeks. All participating women included in the study gave written informed consent for the use of ultrasound images and clinical data. All the methods hereby explained were performed in accordance with the relevant guidelines and regulations and approved, together with the study protocol, by the ethics committee of the Obstetrics and Gynecology Hospital Affiliated to Fudan University (2018-73). Of these, 731 babies with GA 28^+3^–37^+6^ weeks were delivered within 72 h after ultrasound examination in the hospital. According to the same enrolment criteria of previous studies, the final cohort comprised 295 women with singleton pregnancy, with a total of 295 fetal-lung ultrasound images. The flowchart for the study population is shown in Fig. 3. Gestational age was determined by last menstrual period and verified by first-trimester dating ultrasound (crown–rump length).

Pregnancy complications included GDM and PE. GDM was diagnosed using a 75-g oral glucose tolerance test at 24–28 weeks of gestation^27^. Pre-eclampsia and gestational hypertension are characterized by the new onset of hypertension (> 140 mmHg systolic or > 90 mmHg diastolic) after 20 weeks gestation^28^.

Analysis of neonatal clinical data was supervised by a neonatal doctor. NRM included respiratory distress syndrome (RDS) or transient tachypnea of the newborn (TTN). The diagnosis of RDS and TTN is based on symptoms, signs and radiological examination^7,29^. Diagnostic criteria of RDS: tachypnea, snoring, chest wall retraction, nasal dilatation, the need for supplemental oxygen and the appearance of chest X-rays led to admission to the neonatal intensive care unit for respiratory support. Diagnostic criteria of TTN: mild or moderate respiratory distress (isolated tachypnea, rare snoring, slight retraction) and a chest X-ray (if done) showing alveolar and/or pulmonary interstitial effusion and prominent pulmonary vascular patterns.

### Ultrasound imaging and segmentation

All ultrasound images were obtained during routine prenatal ultrasound examinations within 72 h before delivery. Among which, the images of the training set were obtained by radiologist 1 with more than 10 years’ experience in obstetric and gynecological ultrasound imaging, using aWS80A ultrasound system (Samsung, Korea). The frequency of the CA1-7A probe was 1–7 MHz, with a center frequency was 4.0 MHz. The images of the testing set were obtained by radiologist 2 with 3 years’ experience in obstetric and gynecological ultrasound imaging, using a VOLUSON E8 ultrasound system (GE, United States) . The frequency of the C1-5-D probe was 2–5 MHz, with a center frequency was 3.5 MHz.

**Figure 4:**
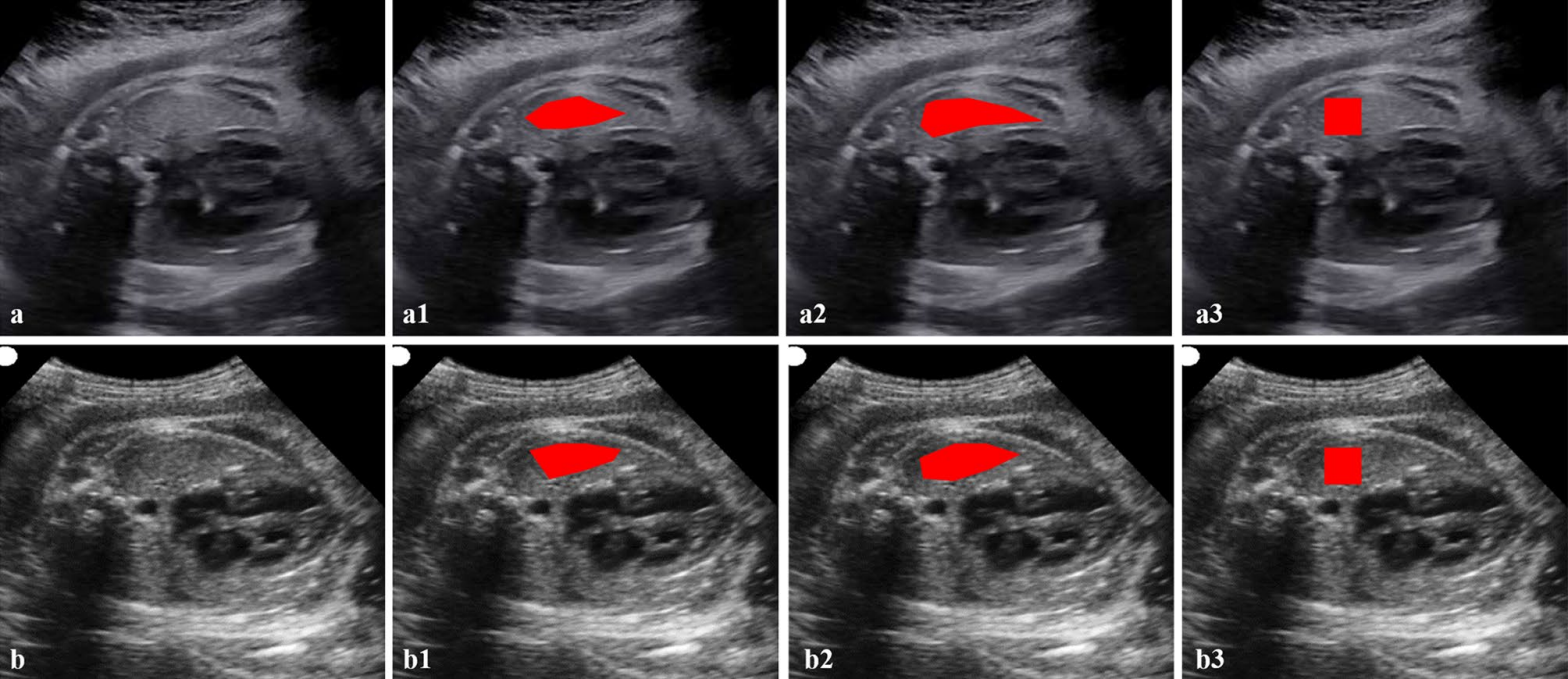
Fetal human lung ultrasound images with defined regions of interest. (**a,a1,a2,a3**) Are images of training set. (**b,b1,b2,b3**) Are images of testing set. (**a1,b1**) Manual delineation (radiologist **A**) of each lung. (**a2,b2**) Manual delineation (radiologist B) of each lung. (**a3,b3)** Square delineation (40 × 40 pixels) of each lung. (**a,a1,a2,a3**) Image of left lung at 36^+1^ weeks in woman with pre-eclampsia (PE). Cesarean delivery occurred 2 days after ultrasound examination, and baby was diagnosed with transient tachypnea of the newborn. The risk probability derived from the model is 0.829 (> 0.5). (**b,b1,b2,b3**) Image of left lung at 34^+0^ weeks in woman with gestational diabetes mellitus (GDM). Cesarean delivery occurred immediately after ultrasound examination, and baby was diagnosed with respiratory distress syndrome. The risk probability derived from the model is 0.843 (> 0.5).

A detailed description of the standard image acquisition protocol and the method used of manual (free-hand) delineation is fully described in a previous study^25^: Briefly, the standard fetal lung images requiring: on an axial section of the fetal thorax at the level of the four-chamber cardiac view, the settings were adjusted (depth, gain, frequency and harmonics) to ensure that at least one of the lungs had no obvious acoustic shadowing from the fetal ribs. All the images were inspected for image quality control and stored in DICOM format (.dcm) for offline analysis. Manual (free-hand) delineation was performed in each fetal lung by two radiologists (radiologists A and B), and square delineation (40 × 40 pixels) was performed by radiologist B, selecting one side of the fetal lung, taking great care to ensure that only the lung tissue was delineated, and avoiding blood vessels, rib shadows, and the lung capsule, as shown in Fig. 4. The radiologist A’s segmentation results were used to generate the model, while the radiologist B’s segmentation and the square delineation results were utilized to verify the stability of the model.

### Radiomics evaluation and machine learning

**Figure 5:**
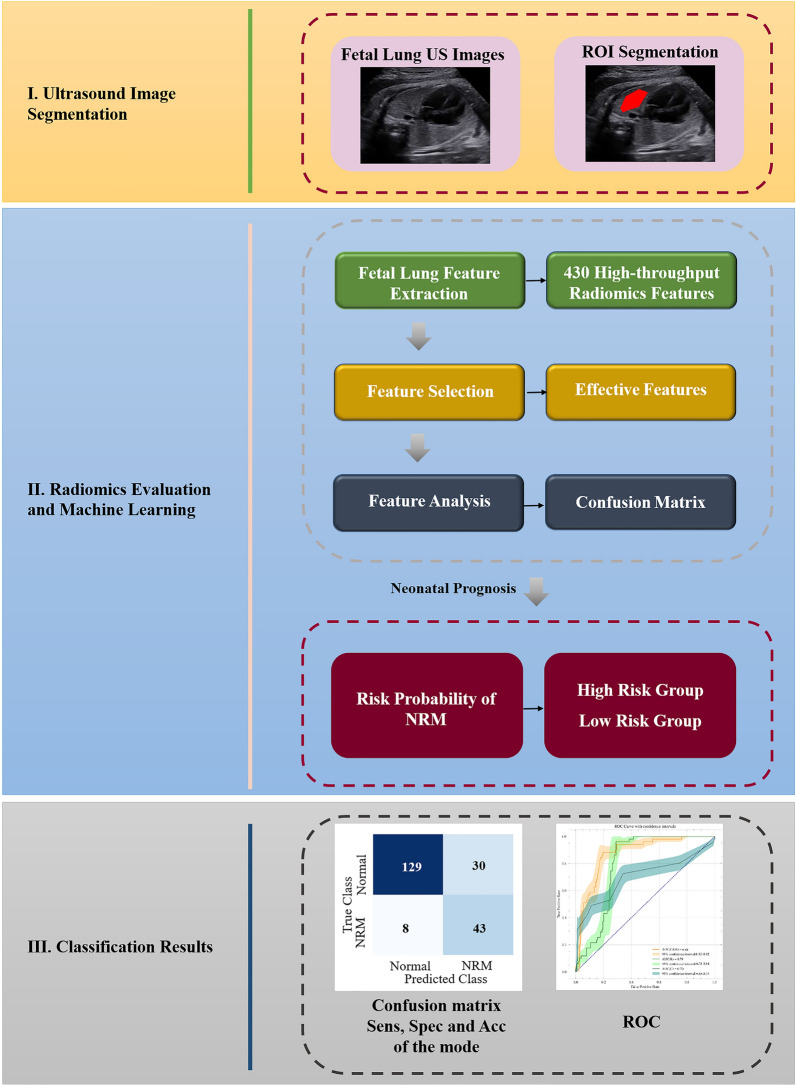
Workflow of the fetal lung texture analysis system based on ultrasound-based radiomics technology. Stage I: Fetal-lung US image (four-chamber view) was segmented manually. Stage II: 430 high-throughput radiomics features were extracted from each segmented image. Then features were selected by permuting out-of-bag data feature of random regression forest. And the prediction model was built using RUSBoost (Random under-sampling with AdaBoost). Finally, the risk probability of NRM in each fetal lung image was obtained and divided into the high-risk group or low-risk group. Stage III: According to results of confusion matrix, performance of prediction model was assessed by sensitivity (SENS), specificity (SPEC), accuracy (ACC) and area under receiver-operating-characteristics (ROC) curve. *ROI* Region of interest, *US* ultrasound, *NRM* neonatal respiratory morbidity, *Sens* sensitivity, *Spec* specificity, *Acc* accuracy, *ROC* receiver operating characteristics.

The research process is shown in Fig. 5.

All the feature extraction and image classifications were carried out using Matlab R2018a and Toolbox Classification (Mathworks, Inc, Natick, Massachusetts, US).

Univariate analysis was used to describe the differences in features among the different categories. The t-test was performed on each 430 continuous radiomics features^25^, including 15 morphological, 73 texture and 342 wavelet features. The χ^2^ test was performed for two categorical clinical features, gestational age and pregnancy complications. *P* value < 0.05 indicated a significant difference.

The feature extraction method to analyze each ROI has been previously reported^25^. First, high-throughput radiomics features importance per fetal lung image were ranked to selected features by permuting out-of-bag data feature of random regression forest. If a feature is influential, permuting its values would influence the model error testing with out-of-bag data. The more important a feature is, the greater its influence will be^30^. As a result, 20 radiomics features (2 texture features and 18 wavelet features) and 2 clinical features (GA and Pregnancy complications) were selected to classification, which are shown in Table 4. The stability of selected radiomic features depending on different delineations (manual delineation by radiologists A and B and square delineation) was analyzed with ICC (2, 1)^31^. Then, the diagnostic performance of predicting neonatal respiratory morbidity depending on different features was compared, including clinical features (GA and pregnancy complications), radiomics features and the combination of clinical and radiomics features. For clinical features, a support vector machine (SVM) classifier was used for classification. By adjusting the cost of misclassification in different categories, the classifier can focus on the positive samples. For radiomics features and the combination of clinical and radiomics features, with the high imbalance of samples and the small sample size, RUSBoost (Random under-sampling with AdaBoost)^32^ was used to build the model. Finally, the risk probability of NRM in each fetal lung image was obtained, which was the predicted score normalized to the range of 0–1 by softmax function of the RUSBoost. The cut-off point of the model was 0.5. The fetal lungs with risk probability higher than 0.5 were divided into the high-risk group, and lower than 0.5 were divided into the low-risk group. All classifier parameters were tuned with bootstrap tenfold cross-validation, and the decision tree was employed as the base learner for RUSBoost.

**Table 4:** List of high-throughput sonographic features.

Feature type (n)	Feature name	*P* value	ICC (95% CI)
Clinical features (2)	GA	0.000	–
	GDM or PE	0.002	–
Texture features (2)	Run-length variance	0.014	0.933 (0.906–0.954)
	Short-run high grey-level emphasis	0.061	0.932 (0.905–0.953)
Wavelet features (18)	Skewness of diagonal	0.001	0.820 (0.755–0.872)
	Mean of contrast of approximation	0.005	0.846 (0.789–0.892)
	Energy of vertical	0.008	0.945 (0.922–0.962)
	Busyness of diagonal	0.010	0.705 (0.599–0.790)
	Complexity of vertical	0.010	0.903 (0.847–0.938)
	Grey-level variance of diagonal	0.021	0.925 (0.890–0.950)
	Grey-level variance of vertical	0.021	0.961 (0.944–0.973)
	Mean of contrast of diagonal	0.031	0.949 (0.928–0.965)
	Short-run high grey-level emphasis of diagonal	0.036	0.942 (0.908–0.963)
	Variance of vertical	0.041	0.960 (0.943–0.973)
	Variance of approximation	0.055	0.911 (0.875–0.938)
	Mean of covariance of diagonal	0.110	0.952 (0.932–0.967)
	Sum entropy of diagonal	0.188	0.937 (0.912–0.957)
	Information measure of correlation 1 of approximation	0.270	0.949 (0.928–0.965)
	Inverse difference moment normalized of approximation	0.360	0.913 (0.878–0.940)
	Standard deviation of approximation	0.372	0.937 (0.912–0.957)
	Mean of covariance of horizontal	0.653	0.866 (0.814–0.906)
	Mean of covariance of vertical	0.916	0.948 (0.920–0.966)

Between the normal group and the NRM group, T-test was performed for each feature. *P* value < 0.05 indicated a significant difference. The smaller the *P* value, the greater the probability that the feature is significantly different between the two groups. The stability of selected radiomic features depending on different delineations (manual delineation by radiologists A and B and square delineation) was analyzed with ICC (2, 1).

*GA* gestational age, *GDM* gestational diabetes mellitus, *PE* pre-eclampsia, *ICC* intraclass correlation coefficient.

The prediction performance of the model was assessed for sensitivity (SENS), specificity (SPEC), accuracy, PPV, NPV and AUC.

## Data Availability

The data that support the findings of this study are available at the web repository of https://pan.baidu.com/s/1p9kat4pr3jFrE1jPE8O5wA and its extraction code can be obtained from the corresponding author upon a separate request.
